# A Rare Case of Duodenum Inversum in a Pediatric Patient

**DOI:** 10.1155/crpe/9994811

**Published:** 2025-10-01

**Authors:** Rosstin Ahmadian, Diana R. Cardero, Kristel N. Montaño

**Affiliations:** ^1^Department of Cell Biology and Physiology, University of New Mexico School of Medicine, Albuquerque, New Mexico, USA; ^2^Department of Family Medicine, University of New Mexico School of Medicine, Albuquerque, New Mexico, USA; ^3^Department of Pediatrics, University of New Mexico School of Medicine, Albuquerque, New Mexico, USA

## Abstract

Duodenum inversum is a rare congenital anomaly, where the third portion of the duodenum reverses direction and travels posteriorly and superiorly prior to crossing the midline. This condition is associated with nonspecific symptoms such as abdominal distension, epigastric pain, and nausea and is typically found incidentally. This report discusses the findings of duodenum inversum in a 6-year-old male who presented with recent weight loss, nausea, vomiting, abdominal pain, and physical exam findings concerning for superior mesenteric artery syndrome and ultimately found to have functional abdominal pain.

**Summary**

• This case report describes a complex case of functional abdominal pain confounded by the presence of duodenum inversum in a pediatric patient.

## 1. Introduction

Duodenum inversum is a rare congenital anomaly where the third portion of the duodenum reverses direction and travels posteriorly and superiorly prior to crossing the midline. Although duodenum inversum is of unknown origin, it is thought to result from dorsal mesentery persistence within the mobile duodenum [1]. This rare condition is generally found incidentally in adult patients who present with nonspecific symptoms (e.g., epigastric pain, nausea, or distension); however, a handful of cases have been reported in the pediatric population [2].

## 2. Case Presentation

A 6-year-old male with a past medical history of attention deficit hyperactivity disorder (ADHD) managed with dextroamphetamine–amphetamine and clonidine presented to the pediatric urgent care with a 5-day history of nausea, vomiting, and abdominal pain. The patient endorsed occasional difficulty stooling in the past month, and his last bowel movement was 5 days prior to presentation. Review of systems was negative for chronic nausea or vomiting. Vital signs upon arrival included a blood pressure of 127/87 mmHg (> 95^th^ percentile), heart rate of 81 beats per minute, an oxygen saturation of 95% while breathing ambient air, and a temperature of 36.5°C. The patient had a height of 121.0 cm and a weight of 21.5 kg, which placed him at the 51^st^ percentile for height and 49^th^ percentile for weight (using Centers for Disease Control growth charts). The patient's weight was noted to be down 6.6 kg from 10 months prior. Abdominal examination revealed a soft, nondistended abdomen with mild epigastric tenderness to palpation and no rebound, guarding, or palpable mass. Abdominal pain was attenuated in the prone position and augmented when supine and upright. Laboratory results were within reference ranges including electrolyte panel, liver function tests, and urinalysis.

**Figure 1:**
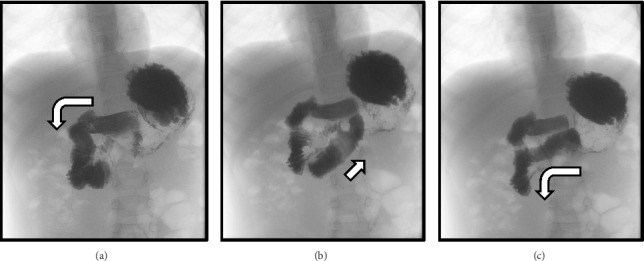
Sequential images from upper gastrointestinal series. Arrows indicate the direction of contrast movement. (a) Normal appearance of the first and second parts of the duodenum. (b and c) The third part of the duodenum ascends with reversal and redundancy of the fourth duodenal part. Following redundancy, the duodenum drains within normal limits.

This patient was initially evaluated for superior mesenteric artery (SMA) syndrome, given isolated epigastric abdominal pain, vomiting, and a recent 6.6 kg weight loss with physical exam findings notable for pain worsening when either supine or upright and pain improvement when prone. Abdominal X-ray (ABD X-ray) revealed a moderate stool burden with no abnormal air-fluid levels, evidence of pneumatosis, portal venous gas, or pneumoperitoneum. No abnormal calcifications or acute osseous abnormalities were appreciated. In addition, a fluoroscopy upper gastrointestinal (UGI) exam series was conducted, which revealed no evidence of proximal duodenal dilation or mid-duodenal obstruction to suggest SMA syndrome. However, a redundancy of the proximal duodenum was identified, suggestive of duodenum inversum (Figure 1). Further abdominal imaging for SMA syndrome was deferred due to the lack of gastrointestinal obstruction seen on imaging.

Due to the presence of moderate stool burden in the setting of abdominal pain, the patient was admitted for polyethylene glycol (PEG) bowel clean-out and rehydration. During admission, the patient experienced multiple bowel movements, resolution of nausea and vomiting, diet tolerance, and improved abdominal pain. Pediatric gastroenterology (GI) and pediatric surgery were consulted during this admission. Surgical intervention was not indicated due to the lack of intestinal obstruction or surgical abdomen in the setting of symptom improvement following the bowel regimen. The patient was discharged after one day. GI outpatient follow-up was scheduled at discharge due to the incomplete resolution of his abdominal pain despite evacuation of the stool burden. Differential diagnoses for this persistent abdominal pain included gastroesophageal reflux disease (GERD) and functional abdominal pain. Discharge medications included scheduled PEG, cyproheptadine, omeprazole, and peppermint oil capsules.

At his follow-up GI appointment, the patient was found to have regular bowel activity with persistent mild abdominal pain and recurrence of vomiting. Esophagogastroduodenoscopy (EGD) was scheduled to evaluate for mucosal lesions secondary to GERD. This demonstrated anatomy within normal limits on appearance, and only a small fragment of oxyntic mucosa with mild chronic inactive gastritis was appreciated on pathology. The patient was advised to continue cyproheptadine for functional abdominal pain, PEG as needed, and a high fiber diet. He responded well to the treatment plan and experienced weight gain along with the resolution of symptoms by the 5-month follow-up.

## 3. Discussion

Duodenum inversum is generally found incidentally in pediatric patients who present with GERD, abdominal pain, nausea, vomiting, stiffening, and fussiness (Table 1). Complications as a direct result of duodenum inversum may include gastroesophageal reflux, duodenitis, acute pancreatitis, peptic ulcer disease, or GI obstruction [3, 4, 6, 10]. The incidence of duodenum inversum in the general population remains difficult to ascertain since it is a rare anomaly that is generally benign. However, it remains important to consider diagnostically in chronic GERD or acute GI obstruction.

The presence of duodenum inversum and moderate stool burden added a level of complexity to this patient's initial workup; however, he lacked the anatomical complications that are associated with duodenum inversum. The diagnosis of functional abdominal pain in this patient was supported by incomplete pain resolution following bowel regimen, the lack of alternative causes, and positive response to cyproheptadine. The patient's recent weight loss was most likely due to his recent initiation of dextroamphetamine–amphetamine 8 months prior to presentation at the urgent care. Studies have previously shown an effect of methylphenidate on weight during the first 12 months of treatment with normalization occurring after long-term use [11]. A similar trend was seen in this patient.

Refractory abdominal pain and vomiting in pediatric patients require a broad initial evaluation to rule out emergent diagnoses such as obstruction, SMA syndrome, or intestinal malrotation. Indeed, Rome IV pediatric diagnostic criteria for functional gastrointestinal disorders require that “the symptoms cannot be attributed to another medical condition” [12]. The presence of duodenum inversum can certainly complicate this diagnosis of exclusion as well. A pediatric case report recently described a patient with severe functional vomiting, who was found to have duodenum inversum during workup [8]. Medical management of pediatric patients with functional abdominal pain disorders can be challenging and may include strategies including dietary modifications, psychological interventions, or pharmacologic therapy [13].

## Figures and Tables

**Table 1:** Previously reported cases of pediatric duodenum inversum.

Report	Year	Patient age	Gender	Presenting symptoms	Diagnostic imaging	Treatment
Long et al. [3]	1999	16 y/o	Female	Nausea, vomiting, cramping	UGI	Exploratory laparotomy
Kim et al. [2]	2013	1 m/o	Male	GERD	UGI	Diagnostic laparoscopy
Patel et al. [4]	2016	2 m/o	Male	Fussiness, stiffening	UGI	Nissen's fundoplication
Patel et al. [4]	2016	2 m/o	Male	Stiffening, gasping, spitting up	UGI	Proton pump inhibitors
Dogan et al. [5]	2016	12 y/o	Female	Nausea, vomiting	UGI	Proton pump inhibitors
Menchise et al. [6]	2016	10 y/o	Female	Abdominal pain, bilious emesis	ABD U/S, ABD X-ray, UGI	EGD, nasojejunal tube
Winborne and Sims [7]	2023	7 m/o	Female	Nonbilious emesis	UGI	Famotidine
Sharma et al. [8]	2023	16 y/o	Male	Nonbilious emesis	ABD CT, UGI, ABD X-ray	Amitriptyline, cyproheptadine, and total parenteral nutrition
Srivatsa et al. [9]	2024	13 y/o	Male	Bilious emesis	ABD X-ray, UGI, ABD U/S	Surgical correction

*Note:* UGI, fluoroscopy upper gastrointestinal exam series; ABD U/S, abdominal ultrasound; ABD X-ray, abdominal X-ray; EGD, esophagogastroduodenoscopy; ABD CT, abdominal computed tomography scan.

Abbreviation: GERD, gastroesophageal reflux disease.

## Data Availability

Data sharing is not applicable to this article as no datasets were generated or analyzed during the current study.

## References

[1] Childress M. H. (1979). Duodenum Inversum. *Journal of the National Medical Association*.

[2] Kim M. E., Fallon S. C., Bisset G. S., Mazziotti M. V., Brandt M. L. (2013). Duodenum Inversum: A Report and Review of the Literature. *Journal of Pediatric Surgery*.

[3] Long F. R., Mutabagani K. H., Caniano D. A., Dumont R. C. (1999). Duodenum Inversum Mimicking Mesenteric Artery Syndrome. *Pediatric Radiology*.

[4] Patel D., Agarwal R., Powell W., Al-Ansari N. (2017). Gastro-Oesophageal Reflux Associated With Duodenum Inversum: Two Case Reports and a Review of the Literature. *Paediatrics and International Child Health*.

[5] Dogan M. S., Doganay S., Koc G., Gorkem S. B., Coskun A. (2016). Duodenum Inversum: Findings From an Upper Gastrointestinal Series. *Sultan Qaboos University Medical Journal*.

[6] Menchise A. N., Mezoff E. A., Lin T. K. (2016). Medical Management of Duodenum Inversum Presenting With Partial Proximal Intestinal Obstruction in a Pediatric Patient. *Journal of Pediatric Gastroenterology and Nutrition*.

[7] Winborne L., Sims E. M. (2023). Duodenum Inversum: A Rarely Reported Condition in Children. *The American Journal of the Medical Sciences*.

[8] Sharma V., Heston A. L., Lightwine B., Patel A. (2023). An Anatomic Red Herring Found in the Diagnosis of Functional Vomiting. *Cureus*.

[9] Srivatsa S., Weng Q., Diefenbach K. A., Nwomeh B. C. (2024). Duodenum Inversum as a Cause of Bilious Emesis in a Teenager: A Case Report. *Journal of Pediatric Surgery Case Reports*.

[10] Rozek E. C., Graney C. M. (1951). Duodenum Inversum; A Report of Two Cases. *Radiology*.

[11] Carucci S., Balia C., Gagliano A. (2021). Long Term Methylphenidate Exposure and Growth in Children and Adolescents with ADHD. A Systematic Review and meta-analysis. *Neuroscience & Biobehavioral Reviews*.

[12] Hyams J. S., Di Lorenzo C., Saps M., Shulman R. J., Staiano A., van Tilburg M. (2016). Childhood Functional Gastrointestinal Disorders: Child/Adolescent. *Gastroenterology*.

[13] Thapar N., Benninga M. A., Crowell M. D. (2020). Paediatric Functional Abdominal Pain Disorders. *Nature Reviews Disease Primers*.

